# Factors Affecting Non-sentinel Lymph Node Metastasis in Early-Stage Breast Cancer

**DOI:** 10.7759/cureus.74199

**Published:** 2024-11-22

**Authors:** Zeynep Gül Demircioğlu, Mahmut Kaan Demircioğlu, Canan T Tanık, Sitki Gurkan Yetkin

**Affiliations:** 1 General Surgery, Kars Harakani State Hospital, Kars, TUR; 2 Surgical Oncology, Umraniye Training and Research Hospital, Istanbul, TUR; 3 Pathology, Sisli Hamidiye Etfal Training and Research Hospital, Istanbul, TUR; 4 General Surgery, Sisli Hamidiye Etfal Training and Research Hospital, Istanbul, TUR

**Keywords:** breast cancer, cerb-b2, metastasis, non-sentinel, stage

## Abstract

Objective

Breast cancer most commonly metastasizes to axillary lymph nodes via lymphatic drainage. Today, axillary lymph node surgery is at least as important as primary breast surgery.

In today's breast surgery, supported by chemotherapy and radiotherapy, current approaches are taken to avoid axillary dissection in appropriate patient groups.

In our study, we aimed to evaluate and predict the factors affecting non-sentinel lymph node metastasis in patients with early-stage clinically node-negative breast cancer.

Materials and methods

Data of 65 female patients were analysed retrospectively. Patient's age, preoperative haemoglobin values, tumour localisation, tumour diameter, number of sentinel lymph nodes sent for frozen examination, and tumour pathological features were investigated in terms of the number of non-sentinel lymph nodes and metastatic lymph nodes removed by axillary dissection, and whether it affects non-sentinel lymph node metastasis was investigated by statistical analysis.

Results

In the axillary dissection of patients with positive sentinel lymph node metastasis, the number of metastatic non-sentinel lymph nodes was statistically significantly higher in Cerb-B2-positive patients than in Cerb-B2-negative patients (p=0.046). As the stage progressed, the number of metastatic non-sentinel lymph nodes increased as expected (p<0.001).

Conclusion

In the current axillary surgery, it is a new approach to avoid axillary dissection in appropriate patient groups even if the sentinel lymph node is positive. When planning the surgical treatment of these patients, it should be considered preoperatively that Cerb-B2-positive patients are more prone to axillary non-sentinel lymph node metastasis.

## Introduction

Breast cancer is the most common cancer among women and ranks second after lung cancer in cancer-related deaths [1]. Breast cancer most commonly metastasizes to axillary lymph nodes via lymphatic drainage. Today, minimally invasive breast surgery for breast conservation is accepted by all authorities, and the approach to the axilla is also becoming minimally invasive.

According to the Eighth Edition of the American Joint Committee on Cancer (AJCC) tumour/node/metastasis (TNM) classification of breast cancer staging, N is divided into three groups according to the number of metastatic lymph nodes: pN1, 1-3 positive lymph nodes; pN2, 4-9 positive nodes; and pN3, ≥10 positive lymph nodes [2].

Axillary dissection (AD) is a surgical procedure that may cause many morbidities such as chronic lymphedema, weakness, loss of sensation, and pain in the postoperative period. Therefore, the necessity and extent of AD should be performed accurately, and minimally invasive surgical principles should be adhered to. The American Society of Clinical Oncology (ASCO) makes three important recommendations in its 2017 update on sentinel lymph node biopsy (SLNB) in early-stage breast cancer patients: first, patients with negative SLN metastases do not require AD; second, patients with one or two positive SLNs who are scheduled for breast-conserving surgery should not undergo AD in many cases because they will receive radiotherapy; and third, patients who are scheduled for mastectomy and have SLN metastases should undergo AD [3].

In our study, factors related to patient and tumour characteristics that can predict axillary lymph node metastasis and provide minimally invasive axillary surgery were evaluated.

This study aims to identify tumour and patient characteristics that predict non-SLN metastasis in early-stage, clinically node-negative breast cancer, with the goal of facilitating more tailored and minimally invasive axillary surgery for appropriate patient groups.

## Materials and methods

Before the study, approval was obtained from the Sisli Hamidiye Etfal Training and Research Hospital Local Ethical Committee on January 22, 2019 (approval number: 2235). All female patients who underwent surgery for breast cancer in the general surgery clinic of Sisli Hamidiye Etfal Training and Research Hospital between January 2011 and January 2020 and underwent AD because of positive SLNB during surgery were included in the study. Preoperative clinical or radiological positive axilla, previous breast surgery, metastatic/locally advanced/recurrent disease, modified radical mastectomy (MRM), negative SLN on frozen and paraffin examination, and male gender were the exclusion criteria. Patients who underwent AD because no SLN was found in the axilla were also excluded. Thus, patients with early-stage invasive breast cancer who were clinically and radiologically SLN-negative, but whose intraoperative frozen examination revealed positive SLN, and who underwent AD in the same session were included in the study. Data of a total of 65 female patients were retrospectively analysed. A logistic model was created to describe the relationship between SLN positivity and variables thought to affect non-SLN positivity.

Methylene blue- and/or technetium-99m (Tc-99m)-labelled colloid was used for SLN marking. Periareolar methylene blue labelling was performed on all patients seven minutes before surgery. SLNB was completed before the surgical procedure and sent to the pathology laboratory for frozen examination.

All SLNs were examined intraoperatively (frozen section) and postoperatively (paraffin section) by the medical pathology department of Sisli Hamidiye Etfal Training and Research Hospital. Serial sections and immunohistochemical (IHC) staining were also performed in SLNs in which metastasis could not be detected by routine histopathological methods. All patients with macrometastases in SLN underwent AD in the same session. AD was not performed in seven patients in whom metastasis was not detected on the frozen section but micrometastasis was detected after IHC staining. The primary surgery of the patients was breast-conserving surgery or simple mastectomy, but the patients were not grouped according to this criterion since the axillary evaluation was emphasised.

Patients were analysed according to the following parameters: age, preoperative haemoglobin values, presence of comorbidities, follow-up time, disease-free survival time, tumour localisation (side and quadrant), the time between diagnosis and surgery, tumour diameter, histopathological features of the tumour (tumour type, histological grade, lymphovascular invasion, perineural invasion, tumour necrosis, tumour multicentricity, multifocality, estrogen receptor (ER) and progesterone receptor (PR), tumour necrosis, Ki67, Cerb-B2 (HER2)), number of SLNs sent for frozen examination, number of non-SLNs removed by AD, and number of metastatic lymph nodes. The effect of these parameters on non-SLN metastasis was investigated by statistical analyses.

Tumour size was determined pathologically, not clinically or radiologically (pT1-T2). For the pathological classification of tumour size and SLN metastasis size and for all staging, the criteria stipulated in the Eighth Edition of the AJCC/Union for International Cancer Control (UICC) staging guidelines were used.

For the Ki67 index, groupings were made according to St. Gallen's 2015 breast cancer optimal treatment consensus. For Cerb-B2 (HER2) IHC analysis, (++) and (++++) were considered positive according to ASCO guidelines. ER and PR status were classified according to the Allred scoring guideline.

Statistical analysis

SPSS for Windows, Version 15.0 (Chicago, SPSS Inc.), was used for statistical analysis. Descriptive statistics were given as numbers and percentages for categorical variables and mean, standard deviation, minimum, and maximum for numerical variables. Since the numerical variables did not fulfil the normal distribution condition, two independent group comparisons were made by the Mann-Whitney U test. Comparisons of more than two groups were performed by the Kruskal-Wallis test since numerical variables did not fulfil the normal distribution condition. Subgroup analyses were performed with the Mann-Whitney U test and interpreted with the Bonferroni correction. Relationships between numerical variables were analysed by Spearman's correlation analysis since parametric test conditions were not met. The proportions in the groups were compared by chi-squared analysis. Determinants of numerical variables were analysed by linear regression analysis. The statistical alpha significance level was accepted as p<0.05.

## Results

The mean age of 65 patients was 52.6±10.4 years. While 35 (53.8%) of the patients had cancer in the right breast, 30 (46.2%) had cancer in the left breast. No patient with bilateral breast cancer was found.

When we grouped the patients according to tumour localisation, the most common tumour was found in the upper outer quadrant (35 patients, 53.8% of the patients). Fifty patients (76.9%) were diagnosed with invasive ductal carcinoma, which was the most common tumour (Table 1).

**Table 1:** Distribution of patients according to tumour localisation and histopathological type

		n	%
Quadrant	Upper outer	35	53.8
	Upper inner	7	10.8
	Retroareolar	7	10.8
	Middle outer	6	9.2
	Lower outer	3	4.6
	Lower interior	3	4.6
	Middle inner	1	1.5
	Upper outer+middle outer	1	1.5
	Lower outer+lower inner	1	1.5
	Upper inner	1	1.5
	Upper inner+retroareolar+middle outer	1	1.5
Tumour type	Invasive ductal carcinoma	50	76.9
	Invasive lobular carcinoma	8	12.3
	Invasive ductal+lobular carcinoma (mixed type)	3	4.6
	Invasive micropapillary carcinoma	2	3.1
	Invasive ductal or lobular+micropapillary carcinoma	1	1.5
	Tubular carcinoma	1	1.5

Lymphovascular invasion was found in 50 patients (76.9%) and perineural invasion in 35 patients (53.8%). In 40 patients (61.5%), ductal carcinoma in situ (DCIS) was found to accompany the tumour. Multicentricity was found in nine patients (13.8%) and multifocality in 19 patients (29.2%). Foci of tumour necrosis were observed in 30 patients (46.2%). Fifty patients (76.9%) were positive for ER, and 48 patients (73.8%) were positive for PR. The mean percentage of ER positivity was 56.5±36.0, and the mean percentage of PR positivity was 43.9±35.6. The mean percentage of Ki67 antibody, which is known as a proliferation marker, was 20.9±17.4. Ki67 antibody percentage was found to be less than 14% in 41.5% of the patients and greater than 14% in 58.5%. Cerb-B2/HER2 neu expression was found to be positive in 23 patients (35.4%) (Table 2).

**Table 2:** Detailed histopathological and immunohistochemical features of the tumours DCIS: ductal carcinoma in situ; ER: estrogen receptor; PR: progesterone receptor

	n	%
Lymphovascular invasion	50	76.9
Perineural invasion	35	53.8
Presence of DCIS	40	61.5
Multicentricity	9	13.8
Multifocality	19	29.2
Tumour necrosis	30	46.2
ER presence	50	76.9
PR presence	48	73.8
Ki67 <14%	27	41.5
Ki67 >14%	38	58.5
Cerb-B2 positivity	23	35.4

Patients with clinically negative axilla were included in our study. However, after postoperative pathological examination, the postoperative stage of six patients (9.2%) was updated as stage 3C, and they were evaluated as an advanced-stage disease. Local recurrence was found in two patients (2.6%) during clinical follow-up.

Surgery in 65 patients was also started with SLNB. The mean number of SLNs sent for pathological frozen examination was 2.1±1.1. The mean number of lymph nodes positive for metastasis on frozen examination was 1.6±0.8. The total number of lymph nodes removed in AD was a minimum of 10 and a maximum of 26 with a mean of 14.2±3.2. The mean number of metastatic non-SLNs, which we mainly evaluated in our study, was 1.5±2.5. In 33 patients (50.7%), no metastasis was detected in the non-SLN (Table 3). The mean follow-up period was 17.0±13.6 months. Patients were operated on a mean of 1.9±1.6 months after diagnosis. Mortality was not detected in any patient during follow-up. Disease-free survival was 16.8±13.6 months in all patients.

**Table 3:** Number of lymph nodes in SLNB and AD SLNB: sentinel lymph node biopsy; AD: axillary dissection

	Mean±SD (min-max)
Number of lymph nodes sent to frozen	2.1±1.1 (1-6)
Number of positive lymph nodes on frozen	1.6±0.8 (1-4)
Number of lymph nodes excised in AD	14.2±3.6 (10-26)
Number of reactive non-SLNs in AD	12.5±4.8 (0-26)
Number of metastatic non-SLNs in AD	1.7±2.8 (0-11)

The relationships between numerical variables were analysed by Spearman's correlation analysis since parametric test conditions were not met. There was a statistically significant correlation between the number of metastatic non-SLNs on AD and the number of reactive non-SLNs on AD (p=0.001) (Table 4).

**Table 4:** Factors associated with the number of metastatic non-SLNs according to Spearman's correlation analysis SLN: sentinel lymph node; AD: axillary dissection; ER: estrogen receptor; PR: progesterone receptor

	Number of metastatic non-SLNs	
	rho	p
Age	0.019	0.884
Pre-op haemoglobin	-0.092	0.467
Maximum tumour diameter	0.033	0.794
ER positivity	0.075	0.551
PR positivity	-0.059	0.638
Ki67	-0.184	0.153
Follow-up (month)	0.177	0.168
Disease-free survival (months)	0.156	0.227
Time between diagnosis and surgery (month)	0.152	0.246
Number of lymph nodes sent to frozen	0.130	0.304
Number of positive lymph nodes on frozen	0.194	0.121
Number of lymph nodes excised in AD	-0.172	0.172
Number of reactive non-SLNs in AD	-0.557	<0.001

The number of metastatic non-SLNs in the AD of Cerb-B2-positive patients was statistically significantly higher than Cerb-B2-negative patients (p=0.046). No significant difference was found in terms of axillary non-SLN metastasis when the patients were divided into groups according to Ki67 antibody percentage as less than 14% and greater than 14%. There was a statistically significant difference in the mean number of metastatic non-SLNs in the stage groups (p<0.001). As expected, the number of metastatic non-SLNs in advanced-stage patients was statistically significantly higher than in early stages (p<0.001) (Table 5).

**Table 5:** Factors affecting metastatic non-SLN SLN: sentinel lymph node; DCIS: ductal carcinoma in situ; ER: estrogen receptor; PR: progesterone receptor

		Number of metastatic non-SLNs			
		Mean±SD	Min-max	Median	p
Side	Right	1.66±2.65	0-10	0	0.898
	Left	1.73±2.92	0-11	0.5	
Quadrant	Upper outer	1.61±2.88	0-11	0	0.403
	Other	1.79±2.64	0-9	1	
Tumour type	Invasive ductal carcinoma	1.92±3.05	0-11	0	0.959
	Invasive lobular carcinoma	0.63±0.52	0-1	1	
	Invasive ductal+lobular carcinoma	1.67±2.08	0-4	1	
	Invasive micropapillary carcinoma	1.50±2.12	0-3	1.5	
	Invasive ductal or lobular+micropapillary carcinoma	1			
	Tubular carcinoma	0			
Histological grade	1	0.50±0.55	0-1	0.5	0.816
	2	1.63±2.49	0-10	0.5	
	3	2.14±3.48	0-11	0	
Nuclear grade	1	0.60±0.55	0-1	1	0.858
	2	1.55±2.59	0-10	0	
	3	2.03±3.12	0-11	0	
Lymphovascular invasion	Negative	1.67±3.04	0-10	0	0.619
	Positive	1.70±2.70	0-11	0.5	
Perineural invasion	Negative	1.33±2.51	0-10	0	0.350
	Positive	2.00±2.95	0-11	1	
Presence of DCIS	Negative	2.16±3.10	0-9	0	0.569
	Positive	1.40±2.51	0-11	0	
Multicentricity	Negative	1.86±2.91	0-11	0.5	0.289
	Positive	0.67±1.12	0-3	0	
Multifocality	Negative	1.33±2.38	0-9	0	0.081
	Positive	2.58±3.42	0-11	1	
Tumour necrosis	Negative	1.63±2.49	0-9	0	0.909
	Positive	1.77±3.08	0-11	0	
ER	Negative	2.00±3.25	0-9	0	0.859
	Positive	1.60±2.62	0-11	0.5	
PR	Negative	1.82±3.09	0-9	0	0.759
	Positive	1.65±2.66	0-11	0.5	
Cerb-B2	Negative	1.19±2.32	0-11	0	0.046
	Positive	2.61±3.27	0-10	1	
Ki67	<14%	1.93±3.01	0-10	1	0.634
	>14%	0.53±2.59	0-11	0	
Stage	Stage 2A	0.47±0.72	0-2	0	<0.001
	Stage 2B	0.47±0.86	0-3	0	
	Stage 3A	2.83±1.90	0-6	3.0	
	Stage 3C	9.00±1.41	7-11	9.0	
Advanced stage	No	0.95±1.46	0-6	0	<0.001
	Yes	9.00±1.41	7-11	9.0	

## Discussion

According to the AJCC TNM staging system, it is known that the stage progresses with increasing nodal involvement. Therefore, as expected, in our study, the increasing number of lymph node metastases in terms of metastatic non-SLN was found to be statistically significant in stage 2A compared to stage 2B and in stage 2B compared to stage 3A (p<0.001 for each). The increase in non-SLN metastasis with the progression of the stage shows the consistency of our study.

In our study, AD was performed as a result of positive SLN, and the mean number of positive non-SLNs in the axilla was 1.7±2.8 (minimum 10, maximum 26 lymph nodes). In 33 out of 65 patients (50.7%), no non-SLN metastasis was detected in the axilla, and all lymph nodes were reported as reactive. While 50.7% of the patients had no metastasis other than SLN in the axilla, approximately the same rates were observed when similar studies were analysed in the literature. In the study published by Zhou et al., 60.78% and, in the study published by Gülben et al., 52% of the patients were negative for non-SLN metastasis. Therefore, it is debated whether AD is actually an "extra treatment". In most cases, there is no metastasis in the axilla except for the first positive SLN or less than three positive lymph nodes [4].

When grouped according to histopathological subtypes, univariate analysis showed that cancer histopathology was not statistically significant on axillary non-SLN metastasis (p=0.959). Similar to our study, Öz et al. and Wang et al. found that cancer histopathology had no effect on non-SLN metastasis [5,6].

About 76.9% of the tumours were positive for lymphovascular invasion. Lymphovascular invasion is an issue emphasised by many researchers in the literature. In their study published in 2018 with 1125 patients, Zheng et al. found that tumour diameter, lymphovascular invasion positivity, and HER2 overexpression were independent factors associated with non-SLN metastasis [7]. Some studies have developed online databases that calculate the probability of non-SLN metastasis. In the database shown in the relevant reference, it predicts that non-SLN metastasis can be predicted using only three parameters. These are lymphovascular invasion, tumour diameter, and diameter of the metastatic SLN. Researchers have statistically explained that lymphovascular invasion has a predictive power on non-SLN metastasis when used together with tumour diameter, although not alone. In our study, there was no correlation between lymphovascular invasion and non-SLN metastasis (p=0.619). Since SLN diameter was not specified in the pathological examinations in our study, this parameter could not be evaluated [8].

In their patient series published in 2019, Lale et al. investigated the factors affecting non-SLN metastasis in patients with 1 or 2 positive SLNs. Unlike our series, they found lymphovascular invasion, extracapsular spread, and the diameter of the metastatic SLN to be statistically significant [9].

The similarities of this study with our series are that Cerb-B2-positive patients with a high Ki67 ratio are at risk for non-SLN metastasis. Among the molecular subtypes, Cerb-B2-positive patients had a higher rate of non-SLN metastasis.

The association of Cerb-B2 oncoprotein amplification or overexpression with poor prognosis has been described in the literature. However, this poor prognosis seems to be favourable in advanced-stage disease with the possibility of treatment with anti-HER2 antibodies. Since the patients in our study were clinically early-stage breast cancer patients, Cerb-B2 expression gained importance. If Cerb-B2 is found positive in patients with early-stage breast cancer, it should be kept in mind that these patients may be associated with axillary metastasis and poor prognosis [10,11]. In pathological examinations, HER2 was reported as Cerb-B2, and HER2 +2 and +3 were considered positive by IHC examination. Confirmation was performed with fluorescence in situ hybridization (FISH) analysis in suspicious cases. Cerb-B2 was found to be positive in 23 of our patients (35.4%) and was found to have a statistically significant higher risk of axillary non-SLN metastasis compared to Cerb-B2-negative patients (p=0.046). In the related reference, Cerb-B2 overexpression was found to be an effective independent factor on axillary lymph node metastasis, which is the issue we emphasised in our study. Especially in ER- and PR-negative breast cancers, Cerb-B2 positivity was found to be a strong factor that could predict axillary metastasis as tumour stage increased [12,13].

In addition, the Ki67 index indicates tumoural proliferation and is a parameter widely used as a prognosis marker. We expect that cells that proliferate at a high rate will also spread at the same high rate. Some studies in the literature also show that Ki67 is closely associated with poor prognosis and axillary spread [8]. According to the general consensus in the literature, patients were divided into two groups as less than 14% and greater than 14% according to the Ki67 index [14,15]. In our study, we did not find a significant correlation between the Ki67 index and axillary non-SLN metastasis. Although it is thought that tumours with high proliferation will also spread rapidly, we did not obtain results supporting this in our study. Since our study group consisted of early-stage breast cancer patients and patients with high Ki67 index were already excluded from the study in terms of receiving neoadjuvant chemotherapy, we think that there is no correlation between Ki67 index and axillary non-SLN metastasis.

The number of patients can be given as an example of the limitations of the study. However, since AD is an increasingly less commonly performed surgery, we may be lacking in the number of patients. 

## Conclusions

With current developments, the treatment planning of breast cancer has changed from radical surgical interventions to "systemic therapy based on tumour biology and characteristics". When the histopathological features of invasive breast cancer and patient characteristics were examined, as expected, non-SLN metastasis was found at a higher rate in patients with more advanced breast cancer. Patients with high Cerb-B2 and advanced stage were statistically significantly higher in terms of non-SLN metastasis.

These findings suggest that integrating axillary surgical management with predictive parameters of non-SLN metastasis may facilitate more tailored and selective treatment modalities for individual patients.
